# The Impact of Respiratory Function on Functionality and Mortality in ALS Patients

**DOI:** 10.3390/jcm14196702

**Published:** 2025-09-23

**Authors:** Ana Cristina de Medeiros Garcia Maciel, Vanessa Regiane Resqueti, Lariza Maria da Costa, Ana Aline Marcelino da Silva, Jéssica Danielle Medeiros da Fonseca, Rayane Grayce da Silva Vieira, Karen de Medeiros Pondofe, Matías Otto-Yáñez, Jordi Vilaró, Rodrigo Torres-Castro, Roberto Vera-Uribe, Giane Amorim Ribeiro-Samora, Danilo Nagem, Ricardo Alexsandro Valentim, Mario Emílio Teixeira Dourado Júnior, Guilherme Fregonezi

**Affiliations:** 1 PneumoCardioVascular Lab/HUOL, Hospital Universitário Onofre Lopes, Departamento de Fisioterapia Universidade Federal do Rio Grande do Norte, Natal 59072970, Brazil; anacristinamgmaciel@gmail.com (A.C.d.M.G.M.); vanessa.resqueti@ufrn.br (V.R.R.); larizamary@hotmail.com (L.M.d.C.); fisioanams@gmail.com (A.A.M.d.S.); jessy_nielle@hotmail.com (J.D.M.d.F.); rayane.vieira.071@ufrn.edu.br (R.G.d.S.V.); karenpondofe@gmail.com (K.d.M.P.); 2 Laboratório de Inovação Tecnológica em Reabilitação, Departamento de Fisioterapia, Universidade Federal do Rio Grande do Norte, Natal 59072970, Brazil; 3 Grupo de Investigación en Salud, Funcionalidad y Actividad Física (GISFAF), Kinesiología, Facultad de Ciencias de la Salud, Universidad Autónoma de Chile, Santiago 7500912, Chile; matiasotto.kine@gmail.com; 4 Facultad de Ciencias de la Salud Blanquerna, GRoW, Investigación Global Sobre Bienestar, Universidad Ramon Llull, 08022 Barcelona, Spain; jordivc@blanquerna.url.edu; 5 Departamento de Fisioterapia, Facultad de Medicina, Universidad de Chile, Santiago 8330111, Chile; klgorodrigotorres@gmail.com (R.T.-C.); robertovera@uchile.cl (R.V.-U.); 6 Departamento de Fisioterapia, Universidade Federal do Rio Grande do Norte, Natal 59072970, Brazil; gribeirosamora@gmail.com; 7 Laboratório de Inovação Tecnológica em Saúde (LAIS), Universidade Federal do Rio Grande do Norte (UFRN), Natal 59072970, Brazil; danilo.nagem@ufrn.br (D.N.); ricardo.valentim@lais.huol.ufrn.br (R.A.V.); 8 Departamento de Medicina Integrada, Universidade Federal do Rio Grande do Norte, Natal 59072970, Brazil; medourado03@gmail.com

**Keywords:** amyotrophic lateral sclerosis, respiratory function, functionality, mortality

## Abstract

**Objective:** To investigate the relationship between respiratory function, functionality, and mortality in amyotrophic lateral sclerosis (ALS) patients and to determine which respiratory parameters show the strongest correlation with functionality and mortality. **Methods:** The study was conducted in Rio Grande do Norte, Northeast Brazil, between January 2018 and December 2023. This was a retrospective cohort, following individuals with ALS who were evaluated at the University Laboratory. **Results:** A total of 74 ALS patients were included in the analysis, with a mean age of 55.7 ± 13.5 years. Most were male (66.2%) and predominantly presented with spinal-onset ALS (51.3%). Respiratory variables (except peak expiratory flow (PEF)) showed a weak but significant inverse correlation with mortality (FVC% predicted (r_pb_ = −0.260; *p* < 0.001), SNIP (r_pb_ = −0.235; *p* = 0.001), MEP (r_pb_ = −0.207; *p* = 0.007), MIP (r_pb_ = −0.198; *p* = 0.009), and PEF% predicted (r_pb_ = −0.156; *p* = 0.013)). When analyzing their correlation with ALSFRS-R, all variables showed a significant positive correlation (ranging from weak to moderate) with functionality. A reduction of one unit in the respiratory variables PEF% of predicted, maximal inspiratory pressure (MIP), and sniff nasal inspiratory pressure (SNIP) increased the risk of death by an average of 300% (OR = 2.99; 95% CI: 2.05–4.35), 2% (OR = 1.02; 95% CI: 1.01–1.03), and 1% (OR = 1.01; 95% CI: 1.00–1.02), respectively. **Conclusions:** Our findings suggest that direct measurements of respiratory function and muscle strength, particularly PEF and SNIP, may serve as more useful markers to guide early interventions such as non-invasive ventilation, thereby improving quality of life and potentially prolonging survival.

## 1. Introduction

Amyotrophic lateral sclerosis (ALS) is a progressive neurodegenerative disease clinically characterized by the degeneration of upper motor neurons in the brain and lower motor neurons in the brainstem and spinal cord and is considered a multisystem and multifactorial disease [1,2]. Studies indicate that the time from the onset of the first ALS symptoms to diagnosis is typically around one year. The diagnosis is established based on the El Escorial criteria. The global incidence of ALS in Europe and the United States is estimated at 1–2 cases per 100,000 people, with a prevalence of 3–5 cases per 100,000 people [3,4,5].

Studies suggest that bulbar or pseudobulbar onset, associated with upper motor neuron involvement [6,7], has been predictive of poor prognosis, and that pulmonary function and overall functionality may be associated with mortality [8,9].

The El Escorial criteria classify diagnostic certainty based on clinical dissemination, not on the severity of the disease. Respiratory involvement depends more on the site of onset, the duration of the disease, and the rate of progression than on the diagnostic category itself [3]. However, there may be some indirect relationships. Patients with ‘Definite ALS’ (generalized involvement) are more likely to have respiratory involvement or to be closer to it, simply because the disease is more clinically advanced. The ‘Possible’ or ‘Probable’ ALS categories may indicate earlier stages of the disease, in which the respiratory muscles have not yet been affected, but this is not guaranteed. Bulbar-onset ALS can meet the El Escorial criteria earlier and is generally associated with a faster respiratory decline, regardless of the category [10].

The time to diagnosis usually lags behind the onset of symptoms. Therefore, by the time a patient is categorized as ‘definite’, respiratory symptoms may already be appearing. The following variables assessed to monitor respiratory progression are not part of El Escorial: forced vital capacity (FVC), sniff nasal inspiratory pressure (SNIP), Oximetry/capnography at night, and need for non-invasive ventilation (NIV) [11].

In addition, respiratory muscle strength has also been proposed as a predictive biomarker for survival or even ventilation-free survival in ALS patients, including maximal inspiratory pressure (MIP), sniff nasal inspiratory pressure (SNIP), forced vital capacity (FVC) and slow vital capacity (SVC), and transdiaphragmatic pressures [12]. Respiratory muscle weakness is associated with decreased chest wall expansion, vital capacity (VC), dyspnea, difficulty communicating, weak cough, and difficulty clearing the airway [13]. Measurements of FVC and SVC are valid and reliable and strictly adhere to international standards as well as specific ALS guidelines [14]. Additional tests, including SNIP and peak cough flow (PCF), are used in clinical practice to guide interventions [15] and have been used in clinical trials [16]. Despite the significant impact of ventilatory support in improving survival in these patients, the disease inevitably progresses to respiratory failure [8].

The diaphragm, as the primary muscle responsible for respiration, is significantly affected in amyotrophic lateral sclerosis (ALS). Contrary to the notion of it being more resistant, recent studies indicate that the diaphragm is particularly vulnerable to both hypoxia and disease progression in ALS patients. Research utilizing diaphragm ultrasound has demonstrated that ALS patients exhibit reduced diaphragm excursion and thickening fraction, even when respiratory symptoms are minimal. These impairments correlate with decreased vital capacity and increased daytime CO_2_ levels, suggesting early involvement of the diaphragm in the disease process [17].

Histopathological analyses have revealed atrophy in both slow-twitch and fast-twitch muscle fibers of the diaphragm in ALS patients. This atrophy occurs even when overall respiratory capacity is only mildly reduced, indicating that the diaphragm does not possess a unique resistance to ALS-related degeneration [18].

Severe diaphragm weakness in ALS leads to hypoxemia and carbon dioxide retention, as accessory respiratory muscles cannot compensate adequately. This underscores the diaphragm’s susceptibility to hypoxic conditions resulting from its own dysfunction [19].

Using multivariate modeling, it was previously shown that each of the measures (FVC, SVC, SNIP, and PCF) showed a decline over time, with a differential decline in patients with bulbar and spinal onset, more clearly demonstrated in SNIP and PCF than in FVC and SVC [20]. It was found that although FVC and SVC were strongly correlated, SNIP was only moderately correlated with FVC and SVC, reflecting that this test assesses a different aspect of respiratory function [13]. Recently, a multicenter observational study analyzed the decline of commonly used respiratory measures such as FVC, SVC, SNIP, PCF, and the respiratory subscore of the ALS Functional Rating Scale (ALSFRS-R) over time. It was observed that respiratory function declines more rapidly in women with ALS compared with men when measured by FVC, SVC, SNIP, or PCF, but not by the ALSFRS-R respiratory subscore [21,22].

In addition, certain non-pulmonary factors, including sex, age, short time from symptom onset to diagnosis, and bulbar presentation, are associated with decreased survival, making pulmonary function tests (PFTs) appropriate predictors of mortality in these patients [23]. Previous studies have shown that ALSFRS-R and FVC are weakly correlated predictors of survival. When considered together, they synergistically predict survival [24]. A Danish cohort aimed to test the progression rate (ΔFS) of ALSFRS-R as a prognostic marker for survival and concluded that age at onset and time from symptom onset to diagnosis emerged as independent predictors of survival. The rate of symptom progression may predict survival, whether calculated at diagnosis or later in the course of the disease [25].

Regular monitoring of lung function is therefore of great importance. Despite the availability of several pulmonary function tests, none has been considered the best indicator of disease progression and mortality [26]. Some spirometric values have already been described in the literature as survival predictors; a higher SVC at disease onset significantly reduces the risk of death, and a slower rate of decline may reduce the risk of respiratory failure or death, with clinical significance [27].

The impact of lung function on the prognosis of ALS has been widely discussed in the literature. We know that regular monitoring of respiratory function is of great importance. Despite the availability of various lung function tests, none of them has been considered the best indicator of disease progression and mortality. Some spirometric values are already described in the literature as predictors of survival; a higher CVL at the start of the disease significantly reduced the risk of death, just as a slower change in its rate of decline can reduce the risk of respiratory failure or death and be clinically significant [26,27].

The extent of impairment in pulmonary function and respiratory muscle strength, along with functionality throughout disease progression and its correlation with patient mortality, remains unclear. Several studies have clearly established the prognostic value of tracking pulmonary function, with multivariate analyses showing that pulmonary function decline is an independent risk factor for death [22]. Our hypothesis is that respiratory function and muscle strength may be significant predictors of mortality. The main objective of the study was to investigate the association between respiratory function and mortality in ALS patients, identifying which respiratory parameters show the greatest correlation with the clinical outcome of death.

## 2. Materials and Methods

### 2.1. Study Design

This is a retrospective cohort study that followed individuals with amyotrophic lateral sclerosis (ALS) who were assessed at the University Hospital. The study was conducted in Rio Grande do Norte, Northeast Brazil, between January 2018 and December 2023. The study was submitted to and approved by the HUOL Research Ethics Committee (REC), receiving approval number 3.127.064.

### 2.2. Procedures and Data Collection

We included in the analysis men and women over 18 years of age, diagnosed according to the El Escorial criteria, representing both bulbar- and spinal-onset types of the disease. All participants were monitored by a multidisciplinary team at the Neuromuscular Diseases Outpatient Clinic at HUOL/UFRN. We excluded data from participants who did not have recorded pulmonary function test results. The data from lung and respiratory muscle function tests (spirometry, manovacuometry, and SNIP), ALS Functional Rating Scale (ALSFRS-r), and mortality were analyzed.

**Figure 1:**
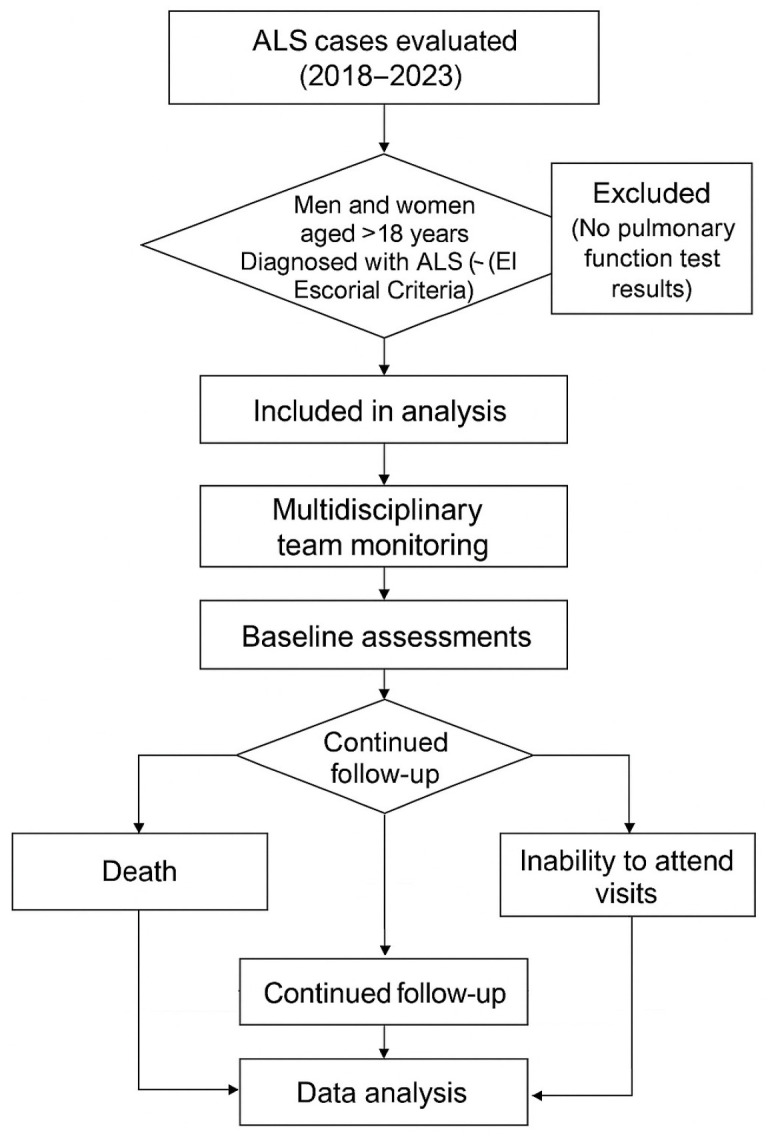
Study flowchart.

Although this is a retrospective study, below we detail the methodology for evaluating patients with amyotrophic lateral sclerosis (Figure 1).

The ALS cases being followed up at the multidisciplinary ALS care center at the Onofre Lopes University Hospital in Natal (Brazil) were collected from 2018 to 2023 using the service’s own database and electronic medical records. Neurologists and general practitioners referred all ALS patients to this center because it is an outpatient center in the national health network and the only ALS treatment center in Rio Grande do Norte. Following diagnosis, patients were monitored at three- to four-month intervals. The same experienced neurologist diagnosed all the patients using the revised El Escorial criteria and collected the following anthropometric and clinical data together with the multi-professional team: age at onset and diagnosis, sex, weight, height, BMI, type of ALS, spirometry and muscle strength values (FVC, FEV1, FEV1/FVC, FEF, PEF, MIP, MEP, and SNIP), and the ALSFRS-R. The collection of these variables occurred at each routine visit, scheduled at three- to four-month intervals, until the patient’s death or inability to attend. Deaths were meticulously documented on dedicated spreadsheets for subsequent analysis. The standardization of data collection was conducted by team members, and the subsequent tabulation of data was undertaken following the provision of prior training.

### 2.3. Spirometry

Spirometry was performed using a Koko Digidoser spirometer (nSpire Health, Longmont, CO, USA) and carried out with the subjects positioned sitting on a chair with feet supported and trunk flexion of 90° according to the ATS/ERS guidelines [13]. All values obtained were compared with the absolute and percentage of predicted values for the Brazilian population [28].

### 2.4. Respiratory Muscle Strength

Maximum inspiratory and expiratory pressures (MIP and MEP) and SNIP were measured using a digital manometer (NEPEB-Labcare, Belo Horizonte, Brazil) with the subjects seated on a chair. MIP was measured starting from residual volume and MEP from total lung capacity, while SNIP was performed starting from functional residual capacity (FRC). Assessments were performed according to ERS recommendations [29,30]. Data obtained were compared with previous reference values [31,32], and the highest value of each test was considered for analysis.

### 2.5. Functionality and Stage of the Disease

Functionality was measured using the ALSFRS-R (maximum 48 points), validated for the Brazilian population [33], as well as its respiratory subscore (R-subscore) alone (maximum 12 points). In addition, the stage of the disease was determined according to disease progression proposed by Roche et al. [34].

### 2.6. Statistical Analysis

The Shapiro-Wilk test was used to verify the normal distribution of quantitative data. As some data did not show a Gaussian distribution, the bias-corrected and accelerated (BCa) bootstrapping method with 1000 replications was used to obtain the respective 95% confidence intervals [35]. Bivariate analyses using Pearson’s correlation test for continuous variables and the point-biserial correlation test for continuous and categorical variables were performed to assess the influence of respiratory variables (MIP, MEP, PEF, SNIP, and FVC) on mortality and disease progression. Age was included as a control variable in all analyses, as it may influence mortality outcomes and functional scale. A generalized linear model (GLM) with binomial probability distribution and logit link function was used to predict mortality. For disease progression (functional scale), normal probability distribution and identity link function were used. In both models, variables with a *p*-value < 0.10 in the bivariate analysis were included, but only those that were statistically significant (*p* < 0.05) remained in the final adjustment. Missing data were treated as missing-at-random. To determine the cutoff point of PEF capable of predicting mortality, an analysis of the area under the ROC curve (AUC) was performed based on the coordinates with the highest sensitivity and specificity. The significance level adopted was an alpha of 5% and the data were analyzed using the Statistical Package for the Social Sciences—SPSS^®^ 25.0 (IBM Armonk, NY, USA) and JASP 0.16.4.0.

## 3. Results

A total of 74 patients were included in the analysis, with a mean age of 55.7 years ± 13.5, the majority being male (66.2%) and predominantly presenting with spinal-onset ALS (51.3%). Table 1 presents the descriptive analysis with the anthropometric and respiratory data of the sample.

The variables associated with mortality were FVC% predicted (r_pb_ = −0.260; *p* < 0.001), SNIP (r_pb_ = −0.235; *p* = 0.001), MEP (r_pb_ = −0.207; *p* = 0.007), MIP (r_pb_ = −0.198; *p* = 0.009), and PEF% predicted (r_pb_ = −0.156; *p* = 0.013). The variables related to the ALSFRS-R were FVC% predicted (r = 0.621; *p* < 0.001), SNIP (r = 0.617; *p* < 0.001), MIP (r = 0.513; *p* < 0.001), MEP (r = 0.511; *p* < 0.001), and PEF% predicted (r = 0.322; *p* < 0.001). These results can be seen in Table 2 below.

The prediction models showed that the MIP and SNIP were associated with both mortality and disease progression; for example, compared with the survivor group, the reduction in PEF% was associated with an almost threefold increase in the odds of mortality (OR = 2.99; 95% CI: 2.05–4.35). Similarly, reductions in MIP and SNIP were associated with 2% (OR = 1.02; 95% CI: 1.01–1.03) and 1% (OR = 1.01; 95% CI: 1.00–1.02) increases in the odds of mortality, respectively (Table 3).

**Figure 2:**
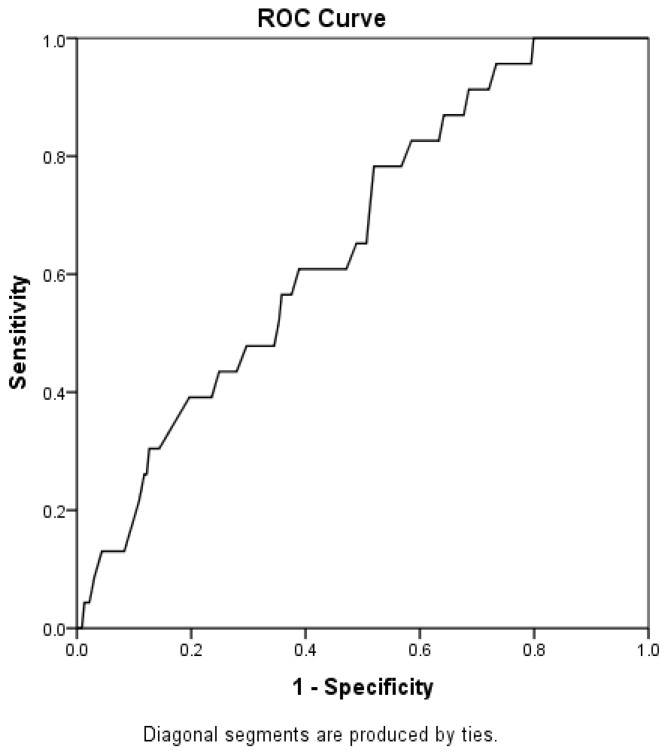
ROC curve.

Considering only the isolated influence of PEF% predicted, we see an even greater contribution to mortality, with an 11.6-fold increase in risk (OR = 11.58; 95% CI: 8.78–15.28). Despite this significant contribution to mortality prediction, it was not possible to establish a cutoff point for PEF, as the AUC was quite low, although still significant (AUC = 0.66; *p* = 0.013; Figure 2). For the worsening of functional status, PEF_%pred_ did not exert a significant influence (*p* = 0.299). However, a reduction of one unit in MIP and SNIP resulted in an average reduction of 0.74 points (95% CI: 0.01–0.14) and 0.20 points (95% CI: 0.10–0.28), respectively, on the functional scale.

## 4. Discussion

The main objective of the study was to investigate the relationship between respiratory function, functionality, and mortality in patients with ALS and to identify which respiratory parameters have the highest correlation with the clinical outcome of death. Most patients with ALS tend to develop a decline in respiratory function, progressive muscle weakness, and paralysis, leading to respiratory failure, which is the primary cause of death. In clinical and experimental trial settings, this decline is commonly assessed by measuring VC, with this data being used to make important patient management decisions. However, understanding the influence of other clinical variables on VC decline, its relationship with functionality, and mortality is clearly important.

Our study expands upon the findings of previous research by investigating in greater detail the association of respiratory variables with functionality and mortality. Our data show that both respiratory function and muscle strength values are associated with functional decline and mortality. The reduction in FVC% predicted, PFE% predicted, MIP, MEP, and SNIP values over time decreases functionality and increases the risk of death.

The studied population was predominantly male (66.2%), with a mean age of 55.7 years and predominance of spinal-onset ALS at diagnosis (51.3%). Population-based studies reveal that ALS affects more men than women, with a ratio of 1.2–1.5:1, and typically occurs in individuals aged 50 to 75 years [36]. Studies in mixed populations, such as the Brazilian population, show disease onset around 55 years [37], which explains the involvement of younger individuals in the country, as observed in our study. This influence of sex and age on the risk of developing ALS is likely due to a neuroprotective effect of endogenous estrogen in women [38].

The ALSFRS-R has been used worldwide as the primary functional outcome scale in ALS. We found that a one-unit reduction in MIP and SNIP resulted in an average reduction of 0.74 points (95% CI = 0.01–0.14) and 0.20 points (95% CI = 0.10–0.28), respectively, on the ALSFRS-R. Although FVC has been the most widely used method for respiratory evaluation in ALS, it requires the patient to expel air rapidly and forcefully, which can cause fatigue, induce bronchospasm, and result in an underestimation of actual pulmonary capacity. Additionally, it presents limitations in patients with advanced bulbar involvement [39]. While functional assessments such as ALSFRS-R and FVC are established markers of disease progression, the prognostic value of MIP and SNIP is less clear. This makes respiratory muscle strength measurements, such as SNIP, an ideal method for use in an outpatient setting, as they can be obtained from patients with advanced disease, minimizing missing data and potentially reducing the underestimation of actual pulmonary capacity due to effort.

In ALS, SNIP is easier to perform than MIP because it does not require a mouthpiece, and its sensitivity may be greater in predicting ventilatory insufficiency; however, both measures are complementary as they reflect different ventilatory mechanics [23]. During SNIP, the effort generated is ballistic, while it is sustained during MIP. Furthermore, the diaphragmatic recruitment pattern is higher in SNIP, resulting in greater transdiaphragmatic pressures. When bulbar involvement is present, the correlation between SNIP and VC is lower, likely due to difficulties in mouth closure, upper airway collapse, and dyspraxia of the upper and lower airways [40]. SNIP is already described in the literature as a predictor of survival [41]; however, this is the first study to quantify that a decline of 1 cmH_2_O in SNIP results in a reduction of 0.2 points on the ALSFRS-R. This demonstrates that maintaining respiratory muscle strength in ALS is directly related to patient functionality and needs to be more closely monitored.

When we analyze the factors associated with mortality, a reduction of 1 cmH_2_O in SNIP and MIP values increases in the odds of mortality by 1% (OR = 1.01; 95% CI: 1.00–1.02) and 2% (OR = 1.02; 95% CI: 1.01–1.03), respectively. Previous studies investigated SNIP as a predictor of mortality by categorizing the measurement into units of 10 cmH_2_O below 50 cmH_2_O, finding that a SNIP ≤ 40 cmH_2_O was 97% sensitive for predicting death within 6 months [39]. It seems reasonable that the ideal clinical practice in ALS for monitoring and potentially initiating ventilatory support which directly impacts functionality and mortality should be based on serial measurements of muscle strength rather than %FVC.

It is important to highlight that a reduction in MIP and MEP could be a modifiable risk factor for respiratory failure, particularly in ALS patients. Due to their small fiber size, abundance of capillaries, and high oxidative aerobic enzymatic activity, diaphragm muscle fibers are resistant to fatigue, while intercostal muscles are less resistant due to their fiber-type composition. However, the structural and functional characteristics of respiratory muscle fibers are not fixed and can be modified in response to training [42]. Progressively monitoring inspiratory pressures with the aim of slowing functional decline through protective measures to maintain muscle strength appears to be indicated.

To explore an even more practical and feasible pulmonary function index for predicting functionality and mortality in ALS, researchers have started emphasizing PEF as a reliable reflection of expiratory muscle function in patients with neuromuscular diseases [43]. Previous studies observed that PEF was an independent predictor of ALS survival and highly correlated with other pulmonary function parameters, including FVC, maximal voluntary ventilation (MVV), FEV₁, and maximum expiratory flow at 75% (MEF75%), demonstrating that PEF was as effective as FVC in reflecting pulmonary function in ALS patients. Additionally, PEF values were moderately correlated with disease severity (assessed by baseline ALSFRS-R scores) and the rate of disease progression (expressed as ΔALSFRS-R) [41]. Our data show that, regarding the worsening of functional status, PEF% predicted did not exert a significant influence (*p* = 0.299). However, in multivariate analysis on the survivor group, the reduction in PEF% was associated with an almost threefold increase in the odds of mortality (OR = 2.99; 95% CI: 2.05–4.35).

Although PEF is different from PCF, PEF is highly correlated with PCF in both healthy individuals and patients with neuromuscular diseases. A cutoff value of 220 L/min for PCF has been suggested for predicting survival [44]. Despite its significant contribution to mortality prediction, it was not possible in our study to establish a cutoff point for PEF because the AUC was very low, albeit significant (AUC = 0.66; *p* = 0.013).

A limitation of this study is that the data were analyzed retrospectively and with a small number of patients. However, the significance found in the univariate and multivariate analyses correlates with functionality and mortality, indicating an obvious clinical utility of frequent muscle strength assessment and PEF as a predictor of mortality in ALS.

Although respiratory muscle weakness is known to be a poor prognostic factor in ALS, there is limited data comparing the prognostic power of different inspiratory and expiratory muscle function tests across various time intervals in any condition. Our data suggest that direct measurement of respiratory muscle function and strength, particularly PEF and SNIP, may serve as more useful markers, aiding in early interventions such as non-invasive ventilation, improving quality of life, and potentially prolonging survival. The current data can also be used to enable smaller sample sizes in future ALS trials, facilitating faster classification of treatments into those with benefit and those without.

## 5. Conclusions

In ALS, we recommend monitoring forced vital capacity (FVC), peak expiratory flow (PEF), maximum inspiratory and expiratory pressures (MIP and MEP), and SNIP to assess respiratory function and disease progression. We included this information in the conclusion section. This study demonstrates that respiratory function, particularly SNIP and PEF values, is closely related to functionality and mortality in ALS patients. Through multivariate analysis, we identified that a reduction in SNIP and PEF significantly increases the risk of death. This suggests that these parameters may serve as more sensitive indicators than FVC for predicting clinical deterioration and the need for early interventions, such as non-invasive ventilation. Additionally, our findings reinforce the importance of routinely assessing respiratory muscle strength, as its decline directly impacts patient functionality and quality of life. Despite the study’s limitations, including its retrospective design and sample size, these results highlight the need to incorporate SNIP and PEF as key monitoring tools in ALS clinical practice. Future prospective studies with larger cohorts will help validate these findings and establish more precise clinical cutoff values to optimize disease management.

## Figures and Tables

**Table 1:** Descriptive analysis of the studied patients.

	Mean (Minimum–Maximum)
Age (years)	55.7 (18–82)
Sex Male/Female (%)	49 (66.2)/25 (33.8)
Weight (kg)	65.6 (39–92)
Height (cm)	164.80 (148–185)
BMI (kg/m^2^)	24.10 (16.23–33.32)
Bulbar type	16 (21.6)
Spinal type	38 (51.3)
Spinal bulbar type	3 (4.1)
N° information of type	17 (23)
FVC (L)	2.99 (0.83–5.23)
FVC_%pred_	0.76 (0.18–1.14)
FEV_1_ (L)	2.26 (0.64–4.22)
FEV_1%pred_	0.70 (0.21–1.08)
FEV_1_/FVC_%_	0.74 (0.32–0.96)
FEV_1_/FVC_%pred_	0.94 (0.49–1.16)
FEF_25–75_	2.74 (0.41–10.04)
FEF_25–75%pred_	0.64 (0.13–1.37)
PEF (L/s)	3.29 (0.25–8.34)
PEF_%pred_	0.46 (0.09–1.28)
ALSFRS-R	37.2 (18.00–47)
MIP (cmH_2_O)	63.4 (8.79–139)
MEP (cmH_2_O)	74.1 (9–163)
SNIP (cmH_2_O)	58 (13–113)

BMI (kg/m^2^): Body Mass Index (kilogram/square meter); FVC (L): forced vital capacity in liters; FEV_1_/FVC_%_: ratio of forced expiratory volume in the first second to forced vital capacity in percentage; PEF (L/s): peak expiratory flow in liters per second; PEF_%pred_: percentage of predicted; ALSFRS-R: Amyotrophic Lateral Sclerosis Functional Rating Scale-Revised; MIP (cmH_2_O): maximal inspiratory pressure; MEP (cmH_2_O): Maximal Expiratory Pressure; SNIP (cmH_2_O) nasal inspiratory pressure. Descriptive statistics expressed as percentage (%), average (minimum–maximum).

**Table 2:** Bivariate associations between respiratory function, mortality, and functionality.

	Mortality		ALSFRS-R	
Variables	r_pb_ (95% CI)	*p* Valor	r (95% CI)	*p* Valor
PEF	−0.10 (−0.20–0.01)	0.110	0.27 (0.15–0.37)	<0.001
PEF_%pred_	−0.16 (−0.23–0.07)	0.013	0.32 (0.21–0.42)	<0.001
SNIP	−0.24 (−0.34–0.12)	0.001	0.62 (0.52–0.69)	<0.001
MIP	−0.20 (−0.32–0.07)	0.009	0.51 (0.38–0.62)	<0.001
MEP	−0.21 (−0.31–0.08)	0.007	0.51 (0.40–0.60)	<0.001
FVC	−0.25 (−0.34–0.12)	<0.001	0.53 (0.43–0.62)	<0.001
FVC_%pred_	−0.26 (−0.39–0.11)	<0.001	0.62 (0.52–0.71)	<0.001

ALSFRS-R: Amyotrophic Lateral Sclerosis Functional Rating Scale-Revised; PEF: peak expiratory flow; SNIP: nasal inspiratory pressure; MIP: maximal inspiratory pressure; MEP: Maximal Expiratory Pressure; FVC: forced vital capacity; %pred.: percentage of predicted. Biserial correlation test (r_pb_) or via Pearson correlation test (r).

**Table 3:** Factors associated with mortality and disease progression.

Dependent Variable	Independent Variable	Coefficient	*p* Valor	OR (CI 95%)
Mortality	PEF_%pred_	1.09	<0.001	2.99 (2.05–4.34)
	MIP	0.02	<0.001	1.02 (1.01–1.03)
	SNIP	0.01	0.001	1.01 (1.00–1.01)
	Constant	0.42	0.42	-
ALSFRS-R	PEF_%pred_	−3.11	0.299	0.04 (0.00–15.64)
	SNIP	0.19	<0.001	1.21 (1.11–1.31)
	MIP	0.07	0.038	1.07 (1.00–1.15)
	Constant	20.22	<0.001	-

ALSFRS-R: Amyotrophic Lateral Sclerosis Functional Rating Scale-Revised; PEF_%pred_: peak expiratory flow as a percentage of predicted; SNIP: nasal inspiratory pressure; MIP: maximal inspiratory pressure. Generalized linear model (GLM) with binomial probability distribution for mortality and normal for ALSFRS-R.

## Data Availability

Data available on request from the authors. The data that support the findings of this study are available from the corresponding author (GAFG) upon reasonable request.

## Abbreviations

The following abbreviations are used in this manuscript:

ALS	Amyotrophic Lateral Sclerosis
PEF	Peak Expiratory Flow
FVC	Forced Vital Capacity
SNIP	Sniff Nasal Inspiratory Pressure
MIP	Maximal Inspiratory Pressure
MEP	Maximal Expiratory Pressure
ALSFRS-R	Amyotrophic Lateral Sclerosis Functional Rating Scale
SVC	Slow Vital Capacity
VC	Vital Capacity
PCF	Peak Cough Flow
PFT	Pulmonary Function Test
HUOL	Hospital Universitário Onofre Lopes
REC	Research Ethics Committee
UFRN	Universidade Federal Do Rio Grande Do Norte
ATS	American Toracic Society
ERS	European Respiratory Society
FRC	Functional Residual Capacity
BCa	Bias-Corrected And Accelerated
GLM	Generalized Linear Model
AUC	Area Under The Curve
BMI	Body Mass Index
FEV1	Forced Expiratory Volume In The First Second
MVV	Maximal Voluntary Ventilation
MEF75%	Maximum Expiratory Flow At 75%
